# A Lover and a Fighter: The Genome Sequence of an Entomopathogenic Nematode *Heterorhabditis bacteriophora*

**DOI:** 10.1371/journal.pone.0069618

**Published:** 2013-07-18

**Authors:** Xiaodong Bai, Byron J. Adams, Todd A. Ciche, Sandra Clifton, Randy Gaugler, Kwi-suk Kim, John Spieth, Paul W. Sternberg, Richard K. Wilson, Parwinder S. Grewal

**Affiliations:** 1 Department of Entomology, The Ohio State University - OARDC, Wooster, Ohio, United States of America; 2 Department of Biology and Evolutionary Ecology Laboratories, Brigham Young University, Provo, Utah, United States of America; 3 Department of Microbiology and Molecular Genetics, Michigan State University, East Lansing, Michigan, United States of America; 4 Department of Genetics, Washington University School of Medicine, St Louis, Missouri, United States of America; 5 Genome Institute, Washington University School of Medicine, St Louis, Missouri, United States of America; 6 Department of Entomology, Rutgers University, New Brunswick, New Jersey, United States of America; 7 Howard Hughes Medical Institute and Division of Biology, California Institute of Technology, Pasadena, California, United States of America; New England Biolabs, United States of America

## Abstract

*Heterorhabditis bacteriophora* are entomopathogenic nematodes that have evolved a mutualism with *Photorhabdus luminescens* bacteria to function as highly virulent insect pathogens. The nematode provides a safe harbor for intestinal symbionts in soil and delivers the symbiotic bacteria into the insect blood. The symbiont provides virulence and toxins, metabolites essential for nematode reproduction, and antibiotic preservation of the insect cadaver. Approximately half of the 21,250 putative protein coding genes identified in the 77 Mbp high quality draft *H. bacteriophora* genome sequence were novel proteins of unknown function lacking homologs in *Caenorhabditis elegans* or any other sequenced organisms. Similarly, 317 of the 603 predicted secreted proteins are novel with unknown function in addition to 19 putative peptidases, 9 peptidase inhibitors and 7 C-type lectins that may function in interactions with insect hosts or bacterial symbionts. The 134 proteins contained mariner transposase domains, of which there are none in *C. elegans,* suggesting an invasion and expansion of mariner transposons in *H. bacteriophora.* Fewer Kyoto Encyclopedia of Genes and Genomes Orthologies in almost all metabolic categories were detected in the genome compared with 9 other sequenced nematode genomes, which may reflect dependence on the symbiont or insect host for these functions. The *H. bacteriophora* genome sequence will greatly facilitate genetics, genomics and evolutionary studies to gain fundamental knowledge of nematode parasitism and mutualism. It also elevates the utility of *H. bacteriophora* as a bridge species between vertebrate parasitic nematodes and the *C. elegans* model.

## Introduction

Nematodes are the most abundant multicellular animals on the planet [1], and exhibit remarkably diverse lifestyles to impact all life [2]. While some nematode parasites harm humans and agriculture, entomopathogenic (i.e., insect-parasitic) nematodes (EPNs) are beneficial in controlling insect pests [3], [4]. Two EPN families, Heterorhabditidae and Steinernematididae, [5], [6] have independently evolved mutual associations with insect pathogenic *Photorhabdus* and *Xenorhabdus* bacteria, respectively [7], [8]. A specialized stage of the nematode, analogous to the *C. elegans* dauer, called the infective juvenile (IJ) harbors the mutualistic bacteria in its intestine while in search of an insect host [9]. Once found, the nematodes penetrate the insect body, sense unknown cue(s) in the hemolymph, and then regurgitate the symbionts [10], [11]. The bacteria grow logarithmically and produce virulence factors and toxins causing rapid insect mortality [12]–[16]. The bacteria produce exoenzymes to degrade the insect tissues and produce unknown metabolites essential for nematode reproduction. Unlike *C. elegans* and other bacteria-feeding nematodes, *H. bacteriophora* reproduces only when associated with specific *Photorhabdus* bacteria both in insects and nutrient rich media [17], [18]. In addition, the *H. bacteriophora* intestine is more permissive to symbiotic and non-symbiotic *Escherichia coli* OP50 intestinal bacteria than *C. elegans* [19]. The bacteria produce potent secondary metabolites that are antibiotics [20] and which deter scavenging arthropods [21], enabling the nematode proliferation to nearly 500,000 IJs from a single infected insect, which then disperse in search of new insect hosts [19], [22].

*Heterorhabditis bacteriophora* and its mutualistic bacterium *Photorhabdus luminescens* represent a model system for the study of symbiosis and parasitism [11], [23], [24]. Although mutually dependent in nature, both organisms can be grown, manipulated and re-associated in culture. *Heterorhabditis* and *Photorhabdus* have congruent evolutionary lineages, indicating significant coevolution [25]. The bacteria adhere, persist, invade and grow inside nematode cells, breaching the alimentary tract to gain access to the developing IJs in the mother’s body [19]. The IJs select for bacteria that adhere to pharyngeal-intestinal valve cells, possibly invade these cells and exit to grow unattached in the intestinal lumen. It is likely that nematode receptors are exposed on specific cells in developmental stages where the bacteria adhere. For example, a phase variant subpopulation of the bacteria express *m*aternal *ad*hesion (Mad) fimbriae required for adhesion to the maternal intestine and transmission to IJs [26]. More surprisingly, the maternal nematodes select for a M-form phenotypic variant that is avirulent and slow growing compared to the insect pathogenic P form [27]. Visualizing the M-form cells persisting in the posterior intestine among the majority transients enabled the discovery that the P form changed to a small cell morphology (i.e. ∼1/7 vol) of the M form. The optical transparency of the nematodes and differential labelling of transient and persistent bacteria made apparent the mutualistic function of phenotypic variant easily ignored. Furthermore, the genetic tractability of the symbiont and ease of screening revealed the mutable locus and transcription factors required for the P and M form switching [26]. It is unknown why nematodes acquire the M form, which switch genetically back to the P form in fully developed IJs and arm these nematodes for insect infection.

The IJs and bacteria endure cooperatively [27], often for many weeks to months without feeding [28] while in search for their host. Lowering their metabolism through cellular acidification and repressed motility may aid the bacteria to persist in the gut of the IJ [27]. In addition to vectoring the bacteria between insect hosts, the IJs may contribute to immune suppression of the insect hosts [29]. Thus, *H. bacteriophora* has evolved sophisticated adaptations for bacterial mutualism enabling it to function as an entomopathogen.

The availability of recent data on genome sequences has laid the necessary foundation for the development of this model system. The complete genome of *H. bacteriophora* strain TT01 symbiont, *Photorhabdus luminescens* subsp. *laumondii* TT01, was released in 2003 [30]. Transcriptomic data of *H. bacteriophora* TT01 and GPS11 recently became available [31]–[33]. Forward genetics by mutagenesis using ethyl methane sulfonate (EMS) was successful [34], [35] [36] and reverse genetics, by gene silencing using RNAi, has been demonstrated in *H. bacteriophora* [24].

Moreover, techniques for genetic diversity assessment [37], [38], genetic selection [39]–[43], hybridization [44], subtractive amplification [45], [46], transcriptional profiling [47], proteomics [48], [49] and DNA transformation [50] have been achieved. Transformation of the *H. bacteriophora* germline with the *C. elegans* heat shock promoter transcriptionally fused to beta-galactosidase [50] and *mec-4* (mechanosensitive) promoter transcriptionally fused to GFP [51] suggest that functional analysis of *H. bacteriophora* genes is possible.

**Figure 1:**
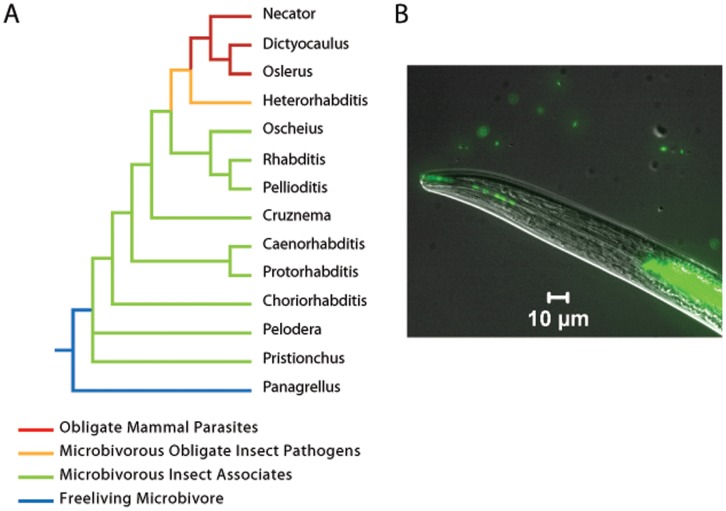
Phylogenetic position of *Heterorhabditis* relative to other notable Rhabditina. **A.** At the base of the tree is the free-living microbivorous *Panagrellus* (Panagrolaimoidea). Lineages in green are semaphoronts of large, diverse clades of microbivorous nematodes whose members associate with invertebrates at some point in their lifecycle, typically via phoresy and/or necromeny [52]–[54]. *Heterorhabditis* is a transitional taxon, exhibiting ancestral microbivorous traits, but has also evolved obligate pathenogensis and shares most recent common ancestry with obligate mammalian parasites (Strongyloidea; lineages in red). Modified after [127]–[129]. Taxonomy follows the ranking hypotheses and nomenclature of Hodda, 2011 [130]. **B.** *H. bacteriophora* nematodes have evolved a mutualism with insect pathogenic *P. luminescens* bacteria (green) where each partner cooperates to achieve voracious entomopathogenicity. An infective juvenile regurgitating intestinal symbionts (right) out the pharynx is shown. The movement of the nematode head causes slight misalignment of the fluorescent and differential interference micrograph image overlays.

Evolutionarily, *Heterorhabditis* is a transitional taxon among the Rhabditina. It exhibits ancestral traits shared with its microbivorous ancestors such as *C. elegans*, but has also evolved parasitism and shares most recent common ancestry with obligate mammalian parasites, such as hookworms and lungworms. Given this phylogenetic position, *Heterorhabditis* can serve as a sort of “bridge” taxon for exploring the evolutionary changes that free-living microbivores have undergone along the path to obligate parasitism of mammals (Figure 1A). Although this figure is not intended to be comprehensive, it does illustrate the general evolutionary trend from free-living microbivory through facultative and obligate associations with invertebrates, to obligate parasitism of vertebrates: *Panagrellus* represents a large clade of free-living microbivores, which gave rise to a series of subsequent evolutionary lineages that are non-parasitic associates of invertebrates, followed by *Heterorhabditis* and its sister taxon, the Strongyloidea (represented by *Necator*, *Dictylocaulus* and *Oslerus*; obligate parasites of vertebrates). According to this scenario, a parsimonious reconstruction of evolutionary history features free-living microbivores giving rise to numerous microbivorous taxa that are facultative or opportunistic associates of invertebrates. However, such facultative and opportunistic conditions gave rise to a clade that evolved obligate parasitism. In *Heterorhabditis* microbivory (Figure 1B) and association with an invertebrate host were maintained. In contrast, the Strongyloidea have lost microbivory during the evolution of obligate parasitism. However, the entomopathogenic symbiosis can also be viewed as an innovation in parasitism where nematode association with an insect pathogen increases the virulence and fitness of insect infection. The clade containing *Dictyocaulus* and *Oslerus* (lungworms; Trichostrongylidae, Metastrongylidae, respectively) has direct lifecycles, being ingested as larvae by their mammalian hosts [52]–[54]. *Necator* (Hookworms; Ancylostomatidae) penetrate tissue to infect its host. Most of the lungworms require an invertebrate (mollusk) intermediate host. Building on this foundation, the objective of this study was to obtain a high quality genome sequence to facilitate further insights into the mutualistic and parasitic lifestyles of *Heterorhabditis*. The analysis of *H. bacteriophora* genome sequence reveals unique features that correspond to the evolution of mutualistic (lover) and parasitic (fighter) aspects of its biology.

## Results and Discussion

A total of 6,845,656 sequencing reads totaling 2,410,251,025 base pairs were obtained from *H. bacteriophora* genome. After quality trimming and assembly, a draft genome consisting of 1,263 scaffolds totaling 77,007,652 bp was obtained. The size of the scaffolds ranged from 327 to 2,228,510 bp with 166 scaffolds larger than 100 kb. The N50 value of the assembled genome is 312,328 bp. The overall GC content is 32.2%, which is similar to the free-living nematode *C. elegans*, plant-parasitic nematode *M. hapla*, and human-parasitic nematode *B. malayi* (Table 1).

**Table 1:** Comparison of *Heterorhabditis bacteriophora* genome with the complete genome of *Caenorhabditis elegans* (WS220) and the draft genomes of *Meloidogyne hapla* [132] and *Brugia malayi* [87].

	*C. elegans*	*H. bacteriophora*	*M. hapla*	*B. malayi*
Life style	Free living	Insect parasitic	Plant parasitic	Human parasitic
Genome size, Mb	100	∼ 80	54	90–95
Scaffolds	n/a	1,263	1,523	8,810
Scaffold N50, bp	n/a	312,328	83,645	93,771
Assembled, bp	100,267,623	77,007,652	53,578,246	70,837,048
Gene models	21,193	21,250	14,420	11,515
Median exon, bp	147	112	145	140
Average exon/gene	6	6	6	7
Median intron, bp	68	125	55	219
G+C, %	35.4	32.2	27.4	30.5

### Protein-coding Genes

The protein-coding genes were predicted using parameters optimized for *C. elegans* in the *ab initio* gene prediction programs. In total, 21,250 protein-coding genes were predicted (Table S1). The majority of the predicted protein genes, 11,207, had no significant homolog to *C. elegans* (WormBase release WS220), whilst 10,043 *H. bacteriophora* proteins had homologs with an E value cutoff of 1e-5 (Table S2). Of the protein-coding genes that have no homologs in WS220, 9,893 had no significant sequence similarity to known proteins in the GenBank non-redundant database and were hence considered novel.

**Figure 2:**
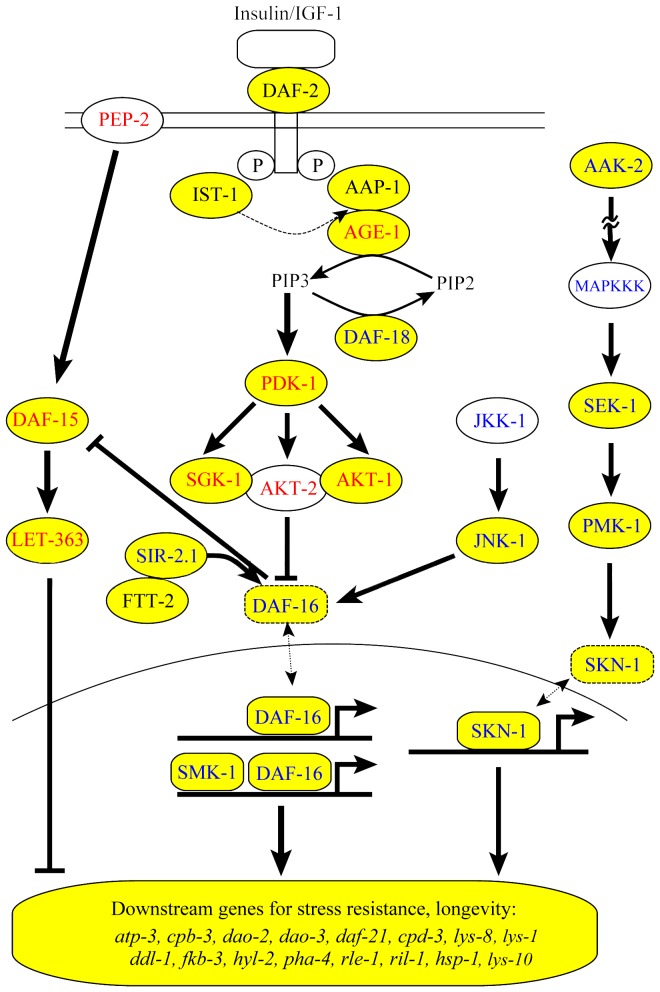
Genes of insulin/IGF-1 signaling pathway in *H. bacteriophora* (highlighted in yellow) and *C. elegans* (all genes). The genes in red and blue fonts are negative regulator and positive regulator, respectively, of stress resistance, lifespan, and immunity in *C. elegan* [131].

*H. bacteriophora* and strongylid parasites like hookworms have adapted a developmentally arrested and alternative third larval stage, known as dauer larva in *C. elegans,* as the infective stage [55]. Entomopathogenic IJs harbor gut symbionts that benefit their insect parasitism [56]. The *C. elegans* dauer develops under stressful conditions such as overcrowding by sensing dauer and other ascaroside pheromones, signal transduction through insulin and TGF-**β** pathways and DAF-12 nuclear hormone receptor [57]–[63]. *H. bacteriophora* produces an ascaroside ethanolamine (C11 EA) derivative that maintains the IJ state at high IJ densities and additional ascarosides [64], [65]. We found that *H. bacteriophora* has most (19 of 23) of the insulin/IGF-1 signaling pathway genes that are critical for dauer formation and for regulation of longevity, stress resistance and innate immunity in *C. elegans* (Figure 2). We also found a *daf-12* homolog predicted to function in ascaroside transcriptional response [66]. Study of IJ formation and exit from diapause, easily tested in insects like *Drosophila melanogaster* and assessed by release of intestinal symbionts [10], [11], may lead to new antiparasitic strategies. Increasing IJ longevity and stress resistance may lead to improvements of EPNs for pest control [28], [67], [68].

**Figure 3:**
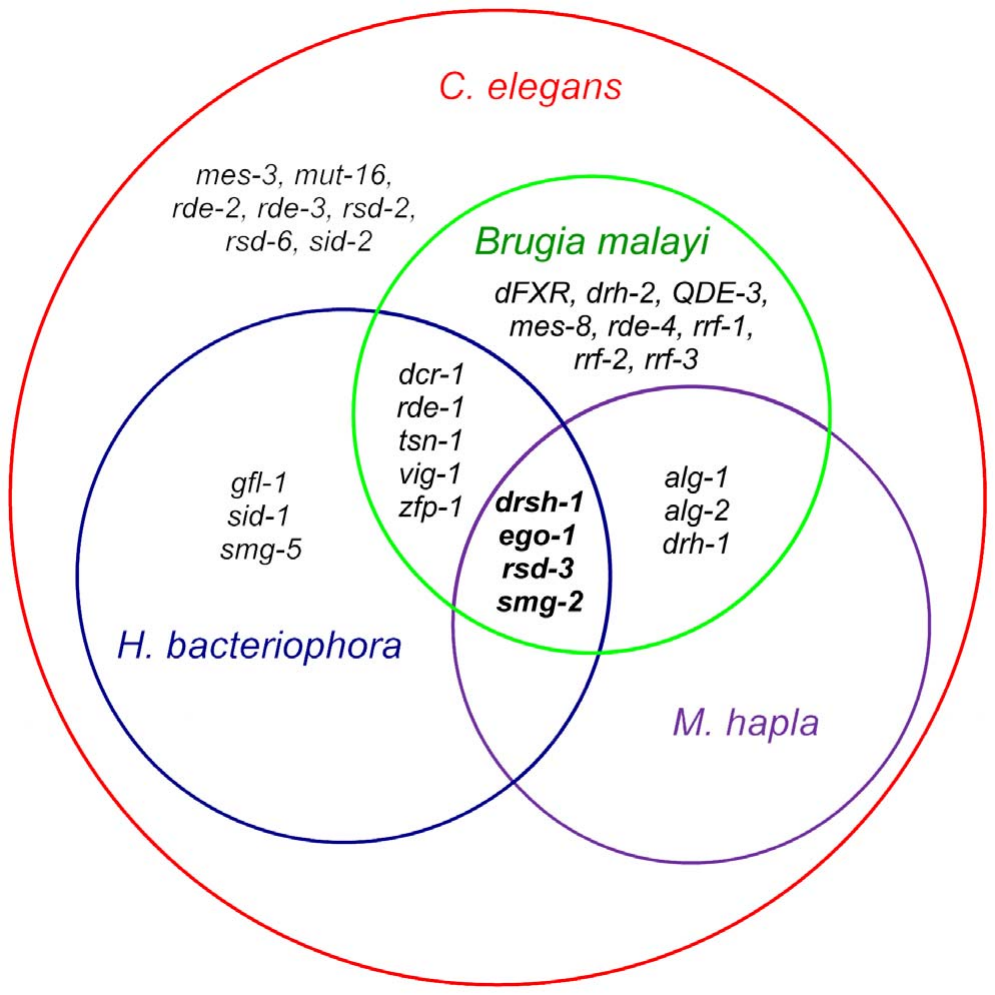
Comparison of genes involved in RNA interference pathway in *C. elegans*, *B. malayi*, *M. hapla*, and *H. bacteriophora*. Four genes in bold, *drsh-1*, *ego-1*, *rsd-3*, and *smg-2* were identified in all four species. *sid-1* gene that is required for systemic RNAi in *C. elegans* was only identified in *C. elegans* and *H. bacteriophora*.

RNA interference (RNAi) is a pathway for gene regulation and powerful tool to manipulate gene expression in functional genomics [69]. RNAi by soaking has been achieved in *H. bacteriophora* [24]. We detected *sid-1* and *sid-3* homologs required for systemic RNAi in *C.* elegans [70], [71] but not a *sid-*2 homolog required in *C. elegans* for the uptake of dsRNA in the intestine [72]. Either an *Hba- sid-*2 homolog was left out of the current *H. bacteriophora* assembly or another transport mechanism is employed. Although *C. elegans* efficiently transports environmental DNA, most other related *Caenorhabditis* species do not [73]. Genes involved in RNA interference in *H. bacteriophora*, *B. malayi*, and *M. hapla* were identified based on sequence similarity to *C. elegans* gene products (Figure 3). Four genes, *drsh-1*, *ego-1*, *rsd-3,* and *smg-2*, have been identified in all four nematode species compared. In *C. elegans*, *drsh-1* gene encodes a predicted RNase III-type ribonuclease that is orthologous to Drosha protein in *Drosophila* and human that is involved in processing primary miRNA transcripts (pri-miRNAs) in the nucleus [74]. *ego-1* gene encodes putative RNA-directed RNA polymerase that is required for germline RNAi [75]. *smg-2* is involved in non-sense-mediated mRNA decay that selectively and rapidly degrades eukaryotic mRNAs with premature stop codons [76]. *rsd-3* is one of four RNA Spreading Defective genes (WormBase). A homolog of *dcr-1* DiCer Related endonuclease [77] was detected in *H. bacteriophora* but not Dcr-1 associated protein *rde-4,* which is required for RNAi in *C. elegans* [78]. Since RNAi has been reported for *H. bacteriophora* [24], *B. malayi* [79], [80], and *M. hapla* [81], different mechanisms are possibly employed.

### Protein Domains

**Figure 4:**
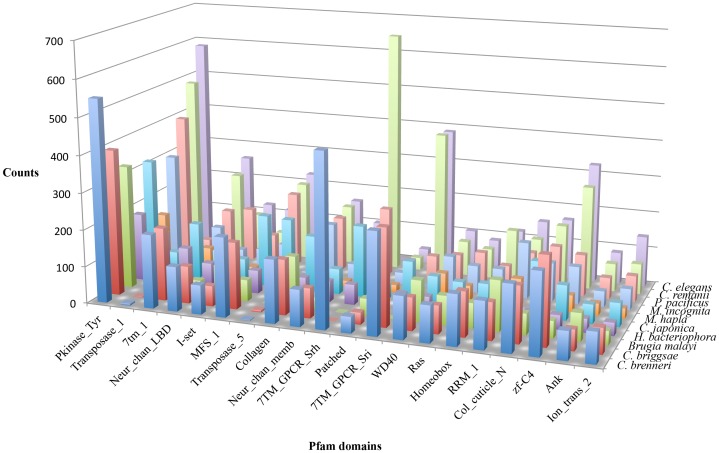
Comparison of top 20 Pfam domains in *H. bacteriophora* genome with those in the 10 nematode species in the study. The top 20 Pfam domains were identified as the ones having the 20 largest number of occurrence in *H. bacteriophora* genome.

To begin to learn how the more than 10,000 unknown proteins function, we analyzed the proteins for conserved domains. A total of 7,957 Pfam domains with 4,144 different Pfam accessions were predicted using the program HMMER [82] with an E value cutoff of 1e-4. We compared the Pfam domains in *H. bacteriophora* with other nematodes [59] (Figure 4; Table S3). Based on protein domain information, we identified 82 members of GPCR (G protein coupled receptor) gene family and 24 members of NHR (nuclear hormone receptor) gene family. The domain richness index analysis (see methods) revealed 56 domains in *H. bacteriophora* that are significantly different from other nematodes. One significantly different richness domain index is the Mariner transposase (PF01359.11), with 138 identified in *H. bacteriophora* proteins compared to 65 in *C. japonica*, one each in *M. incognita* and *M. hapla*, but none in *C. elegans* and *Brugia malayi*. The Mariner transposases have been shown to be sufficient to mediate transposition *in vitro* in a purified form [83]. The enrichment of Mariner transposase domain is in agreement with the 1,314 predicted Mariner DNA motifs that belong to 23 types (Table 2; Table S4). In contrast, a search with the same parameters returned 844 Mariner DNA motifs that belong to 43 types in *C. elegans* genome (Table 2; Table S5). More strikingly, 28 types of Mariner DNA motifs are exclusively present in *C. elegans* genome and 8 types are exclusively present in *H. bacteriophora* genome. The differences in the number and type of Mariner DNA motifs between *H. bacteriophora* and *C. elegans* along with the enrichment of Mariner transposase domains and predicted transposition activity in *H. bacteriophora* is likely evidence of a past or presently mobile genome.

**Table 2:** Numbers of mariner type motifs in *H. bacteriophora* and *C. elegans* genomes.

Mariner type	*Hba*	*Cel*		Mariner type	*Hba*	*Cel*
Mariner2_CE	36	93		Mariner36_CB	39	4
Mariner3_CE	18	73		Mariner37_CB	–	4
Mariner4_CB	2	1		Mariner38B_CB	–	4
Mariner4_CE	27	8		Mariner38C_CB	–	2
Mariner5_CE	4	68		Mariner38_CB	–	1
Mariner7_CB	–	180		Mariner40_CB	–	11
Mariner8_CB	–	6		Mariner41_CB	–	2
Mariner10_CB	–	3		Mariner42_CB	1	2
Mariner12_CB	1	1		Mariner43_CB	8	–
Mariner13_CB	59	9		Mariner44_CB	–	1
Mariner14_CB	135	–		Mariner45_CB	–	6
Mariner15_CB	332	1		Mariner47A_CB	–	14
Mariner16_CB	108	–		Mariner47B_CB	–	9
Mariner17_CB	12	–		Mariner47_CB	–	6
Mariner18_CB	448	–		Mariner48_CB	–	2
Mariner19_CB	12	–		Mariner51_CB	–	2
Mariner20_CB	–	1		Mariner52_CB	–	13
Mariner22_CB	2	4		Mariner53_CB	–	94
Mariner23_CB	–	1		Mariner54_CB	–	17
Mariner25_CB	–	1		Mariner55_CB	1	–
Mariner26_CB	1	–		Mariner56_CB	–	1
Mariner27_CB	1	1		Mariner60_CB	–	4
Mariner28_CB	–	166		Mariner61_CB	–	3
Mariner31_CB	1	10		Mariner65_CB	–	2
Mariner32_CB	64	1		Mariner66_CB	–	4
Mariner34_CB	2	8		**Total**	**1314**	**844**

Abbreviations: Hba, Heterorhabditis bacteriophora; Cel, Caenorhabditis elegans.

We detected far fewer (9 vs. 133) C-type lectin domain-containing proteins than are present in *C. elegans.* Homologs of *lec-1*, *lec-2*, *lec-3*, *lec-5*, *lec-6*, and *lec-12* were detected that function in innate immunity in *C. elegans* [84]. The reduction in C-lectin domain proteins in *H. bacteriophora* may be related to the mutualistic relationship with *P. luminescens* bacteria [19]. Viable symbiotic bacteria are required in the intestine for maternal transmission and in IJs for insect infection. The *H. bacteriophora* intestine is more permissive to symbiotic bacteria and non-symbiotic *E. coli* OP50 than *C. elegans.* Broad-spectrum antibiotics produced by the symbionts likely contribute to defense against pathogenic and saprophyitic microorganisms. *H. bacteriophora* might also contain a diverse and novel set of innate immune effectors that were not detected by homology to *C. elegans.*

### Non-coding RNA (ncRNA) and Regulatory Elements

A total of 134 potential microRNA (miRNA) genes were identified in *H. bacteriophora* genome representing 26 different animal microRNA species (Table S6). Other ncRNA include the U1, U2, U3, U4, U5, and U6 small nuclear RNA (snRNA) components of the spliceosome, SL1 involved in trans-splicing (none if 1e-10 cutoff is used), ribonuclease P (RNaseP), and eukaryotic-type signal recognition particle RNA. The number of the non-coding RNAs detected in *H. bacteriophora* is considerably less than those known to be present in *C. elegans* (Table S6). For instance, *let*-7 is absent in the current assembly although its presence and temporal expression were considered to be conserved among animals with bilateral symmetry [85], possibly due an incomplete genome assembly. The ncRNAs have important roles in regulating transcription, translation, and other biological processes.

A total of 254 transfer RNA (tRNA) genes and 1 tRNA pseudogene were predicted in *H. bacteriophora* genome by tRNAScan-SE (see Table S7) for all 20 standard amino acids, but not the tRNA-Selenocysteine gene. The number of detected tRNA genes in *H. bacteriophora* is dramatically lower than the 659 tRNA genes and at least 29 tRNA pseudogenes in *C. elegans* [86]. However, the number of tRNAs are close to those identified in human and plant parasitic nematodes. There are 233 tRNA genes and 26 tRNA pesudogenes identified in the human parasitic *B. malayi* [87] and 467 tRNA genes, 120 tRNA pseudogenes and 28 other tRNA genes in plant parasitic *M. incognita* [88].

### Microsatellite Repeats

Microsatellites, also known as simple sequence repeats (SSRs), are tandem repeat sequences of 2–6 bp that serve as informative genetic markers to resolve relationships among closely related species because of their high mutation rate [89]. A total of 3,794 microsatellite loci were predicted in 506 contigs of the current draft *H. bacteriophora* genome (Table S8). Among them, 849 were located in coding regions. Previously, we developed 8 polymorphic microsatellite markers for *H. bacteriophora* that distinguished a Northeast Ohio population from other populations [90]. These microsatellite markers can serve as useful tools for determining the phylogeographic, demographic and genetic structure of *H. bacteriophora* populations.

### Estimation of Divergence Time between *H. bacteriophora* and *C. elegans*

The divergence time between *H. bacteriophora* and *C. elegans* was estimated based on a set of 350 orthologs among *H. bacteriophora*, *C. elegans*, *Anopheles gambiae*, and *Homo sapiens*. Based on the divergence time of 800–1000 MYA between nematodes and insects [91], the estimated divergence time between *H. bacteriophora* and *C. elegans* is approximately 86–331 MYA. By contrast, the *C. elegans* and *C. briggsae* speciation date was estimated as 78–113 MYA [91]. The large (conservative) discrepancy between the upper and lower bounds are probably most strongly influenced by the sparse taxonomic sample (n = 4), as well as other analytical biases [92].

### Characterization of the Secretome

*H. bacteriophora* secreted proteins are potentially important for parasitic interactions with insects, mutualistic interactions with symbiotic bacteria, immunity to pathogens and in development and reproduction. We detected 753 proteins with predicted signal peptides of which 150 also were predicted to be membrane localized. The 603 potentially secreted proteins (2.8% of total predicted proteins) are similar to the fraction of *B. malayi* secretome proteins (2.3%), but are less than the free-living nematodes *C. elegans* (10.1%), *C. briggsae* (9.4%), *C. brenneri* (8.9%), *C. japonica* (6.2%), and *C. remanei* (8.8%), and the insect-associated *P. pacificus* (7.4%) when predicted with the same method and criteria. It is also about half of that of plant-parasitic nematodes *M. hapla* (5.2%) and *M. incognita* (5.2%). The low number of predicted secreted proteins in parasitic *H. bacteriophora* and *B. malayi* could be due to their reliance on mutualistic bacteria for these proteins.

Among the 603 *H. bacteriophora* secreted proteins, 164 had significant similarity (E value cutoff of 1e-5) to proteins in the SwissProt database (Table S9). Among the remaining 439 secreted proteins, 122 had significant similarity to proteins in the GenBank non-redundant database. The remaining 317 secreted proteins were novel proteins of unknown function. A search of the MEROPS database containing peptidases and peptidase inhibitors revealed the presence of 1 cysteine, 9 serine, and 9 metallo-peptidases and 9 peptidase inhibitors in *H. bacteriophora* secreted proteins (Table 3). Secreted peptidases have known roles in degrading host tissues for the benefit of parasites [92]. EPNs have been reported to release proteolytic enzymes to aid penetration of the insect gut to reach the hemocoel [93]. Following nematode penetration into the hemocoel, IJ secreted peptides and peptide inhibitors might function to disarm the insect serine proteinase cascade that results in pro-phenoloxidase activation and melanization, the elementary immune defense reaction [94]. However, during subsequent development of the nematode in the host hemocoel, the symbiont secretes peptidases/proteases [13]–[16], [30], which may contribute to such functions. Indeed, the mutualistic bacteria of EPNs also act independently to suppress the insect immune system [29], [95]. Therefore, both partners act synergistically in combating the insect immune system. A peptidase(s) also might function in utilizing symbiont-produced crystalline inclusion proteins (CipA and CipB) that are high in essential amino acid content and required for nematode reproduction [96]. *H. bacteriophora* also has homologs to *C. elegans* lysozyme genes *lys-1, lys-3–8* and *lys-10* that function in bacterial cell lysis and innate immunity [97]. Thus, although similarity suggests common function, it remains to be determined what roles most secreted proteins have in interspecies interactions.

**Table 3:** Summary of secreted peptidases and peptidase inhibitors identified in *H. bacteriophora.*

Protein name	MEROPS family	Query start-end	MEROPS accession	Hit start-end	E value
**Cysteine peptidases**					
Hbpro09515	C46	185–256	MER011696	342–415	1.90e-07
**Metallopeptidases**					
Hbpro17338	M10A	122–237	MER003153	200–317	3.80e-30
Hbpro04992	M12A	92–170	MER003171	124–202	1.60e-20
Hbpro10653	M12A	94–207	MER015241	216–328	6.10e-25
Hbpro13863	M12A	319–441	MER024920	128–246	2.60e-23
Hbpro15592	M12A	133–320	MER002349	134–320	1.10e-91
Hbpro15986	M12A	145–326	MER001107	94–261	1.60e-37
Hbpro20263	M12A	70–253	MER001593	60–237	2.10e-41
Hbpro11857	M12B	104–164	MER002292	347–417	2.00e-25
Hbpro13918	M13	37–327	MER002350	78–370	1.60e-90
**Serine peptidases**					
Hbpro01274	S01A	9–76	MER099499	26–91	2.80e-06
Hbpro17402	S08A	795–954	MER134526	298–451	8.30e-05
Hbpro16490	S08B	203–296	MER001610	179–272	1.20e-52
Hbpro11245	S09X	17–347	MER037861	26–353	1.20e-36
Hbpro11940	S09X	34–208	MER037861	29–207	4.70e-32
Hbpro20894	S10	35–61	MER000430	39–65	3.80e-07
Hbpro12626	S28	59–214	MER162965	54–211	3.50e-34
Hbpro14365	S28	130–242	MER171698	102–212	2.00e-40
Hbpro12626	S37	59–207	MER001350	62–194	1.20e-05
**Peptidase inhibitors**					
Hbpro11626	I02	386–432	MER018193	250–296	4.50e-13
Hbpro12168	I02	20–51	MER022808	669–700	2.90e-09
Hbpro15022	I02	282–333	MER092785	4–53	3.10e-07
Hbpro17931	I02	18–71	MER020231	5–56	1.50e-11
Hbpro06248	I08	21–81	MER017818	10–63	2.50e-05
Hbpro11583	I17	117–167	MER019417	27–69	4.90e-06
Hbpro20975	I21	49–128	MER016218	70–155	5.60e-13
Hbpro11626	I31	325–375	MER020813	331–379	6.10e-08
Hbpro19310	I51	120–189	MER029866	66–135	1.70e-26

### Gene Ontology Enrichment

The predicted Gene Ontology of *H. bacteriophora* proteins was compared to those of the proteins from the other nine sequenced nematode genomes (Table S10). A striking difference is the significant enrichment of DNA metabolic process (GO:0006259), DNA recombination (GO:0006310), DNA-mediated transposition (GO:0006313), DNA integration (GO:0015074), transposition (GO:0032196) and transposase activity (GO:0004803) in *H. bacteriophora* compared to other nematodes, with the exception of *C. japonica*. These observations are in agreement with the enrichment of mariner transposase domain in *H. bacteriophora* discussed above.

### Metabolic Pathway Comparison

The KEGG (Kyoto Encyclopedia of Genes and Genome) pathways were predicted for *H. bacteriophora* and other 9 nematode species for which full genome sequence information is available and the numbers of genes in each pathway are summarized in Table S11. The genes and KEGG orthology (KO) in the metabolic pathways were compared to assess whether there is enrichment or reduction in the *H. bacteriophora* genome compared to other select nematode genomes (Table 4). *H. bacteriophora* has fewer KOs compared to the free-living nematode *C. elegans* in almost all metabolic categories, which is compatible with previous observations that parasitic nematodes seem to undergo reductive genome evolution [98]. However, *H. bacteriophora* has substantially more proteins (48 in total) in the KO groups of glycan biosynthesis and metabolism (Table S12). Glycans are generally found attached to proteins as in glycoproteins and proteoglycans on the exterior surface of cells and play important roles in proper protein folding and cell-cell interactions [99]. At the enzyme level, *H. bacteriophora* has 17 (out of 23) enzymes in common with *C. elegans* (19 enzymes in total). Interestingly, *C. elegans*, *B. malayi* and *M. hapla* have only one isoform (isoform 1) of [heparan sulfate]-glucosamine 3-sulfotransferase (3-OST), whereas *H. bacteriophora* has three isoforms, isoform 1, 2 and 3. The enzyme 3-OST is involved in biosynthesis of glycan structure and different isoforms have been demonstrated to have different substrate specificities depending on the saccharide structures around the modified glucosamine residue [100]. The presence of the two additional isoforms of 3-OST enzyme together with other *H. bacteriophora*-specific enzymes involved in glycan biosynthesis and metabolism suggests that *H. bacteriophora* is well evolved to thrive in different environments where different metabolic substrates are available during its life cycle.

**Table 4:** Genes and KEGG orthology (KO) in metabolic pathways in selected nematode species with different life styles.

Pathway	KOs in KEGG reference pathway	Insect-parasitic *H. bacteriophora*		Human parasitic *B. malayi*		Plant-parasitic *M. hapla*		Free-living *C. elegans*	
		Genes	KOs	Genes	KOs	Genes	KOs	Genes	KOs
Metabolism	2,258	1,224	631	1,694	623	971	572	2,508	790
Amino acid metabolism	484	199	113	196	93	174	102	470	152
Biosynthesis of other secondary metabolites	55	46	22	62	21	32	13	108	25
Carbohydrate metabolism	550	238	145	448	165	207	140	540	199
Energy metabolism	408	124	46	215	51	93	47	207	58
Glycan biosynthesis and metabolism	160	75	48	97	36	43	33	66	35
Lipid metabolism	325	159	85	200	82	137	77	350	99
Metabolism of cofactors and vitamins	301	104	50	130	52	68	40	187	58
Metabolism of other amino acids	126	67	42	55	33	48	36	156	52
Nucleotide metabolism	174	159	49	223	59	104	50	240	68
Xenobiotics biodegradation and metabolism	178	53	31	68	31	65	34	184	44

### Orthologs

The orthologous sequences among H. bacteriophora, C. elegans, C. briggsae, C. japonica, C. remanei, C. brenneri, Brugia malayi, Meloidogyne hapla, M. incognita, and Pristionchus pacificus were identified using the orthoMCL program [101] on the predicted protein sequences from the genomes. In total, we identified 183 orthologs among these species (Table S13). Based on the Gene Ontology information of C. elegans genes in the ortholog sets, most of these orthologs are essential in C. elegans and annotated to biological processes such as reproduction (number of orthologs: 50), growth (36), regulation of growth (47), regulation of biological process (61), and larval development (45). Genome sequences of other nematodes, including Bursaphelenchus xylophilus [102], Trichinella spiralis [98], and Ascaris suum [103], are not included in the analysis because trophic categories represented by these nematodes are already included in the current study.

### *H. bacteriophora* is useful for Comparisons of Rapidly Evolving Protein Domains

**Figure 5:**
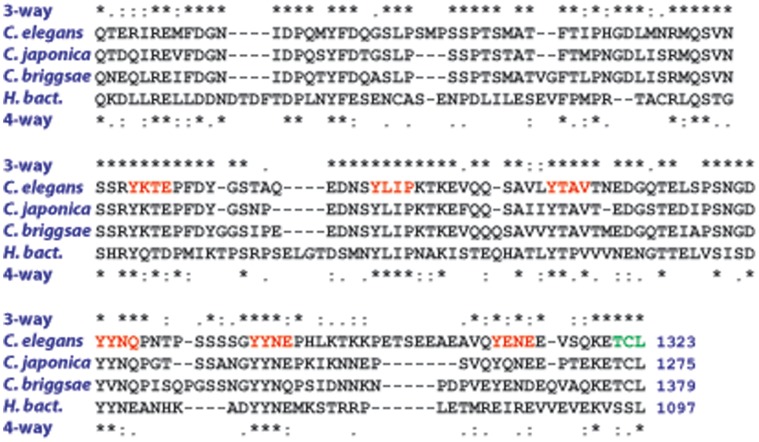
*H. bacteriophora* informs *C. elegans* protein structure function. Multiple alignment of the EGF-receptor (LET-23) carboxyl tail of *Caenorhabditis elegans*, *briggsae* and *japonica* with *H. bacteriophora*. 3-way, alignment of the three *Caenorhabditis* proteins; 4-way, alignment of three *Caenorhabditis* proteins with Hba-LET-23. *, identity; :, strong similarity; ., weak similarity. Red and green highlight the parts of the protein that have been demonstrated to be important in signaling and localization, respectively. Numbers represent the length of the predicted proteins.

Some proteins that are conserved from human to *C. elegans* have domains that are evolving too rapidly to analyze by the large evolutionary distance comparison. One example is the carboxyl terminal tail of EGF-receptor, called LET-23 in nematodes. A three-species comparison of *elegans*-*briggsae*-*japonica* has a C-terminus that is too conserved to be informative (being 65% identical), but addition of *H. bacteriophora* in a 4-way comparison highlights the tyrosines and PDZ-binding domain that have been shown to be functional in LET-23 [104], [105], with only 26% identified across the four species (Figure 5).

### Conclusions

*H. bacteriophora* is an entomopathogenic nematode, which is mutually associated with symbiotic bacteria to function as an insect parasite. The high quality draft genome sequence revolutionizes our knowledge and genetic tractability to understand nematode fundamental processes of gut mutualism and insect parasitism. *H. bacteriophora* is well-known of symbiosis compared to the *C. elegans* and thus represent a simple and tractable model of animal-bacteria gut symbiosis. The genome sequence along with RNAi gene silencing methodology provides a powerful reverse genetic approach to probe the functions of signaling pathways and transcription factors in symbiosis as well as insect parasitism. The *H. bacteriophora* genome sequence along with some sequences from other *H. bacteriophora* strains (e.g. GPS11) allow single nucleotide polymorphisms (SNPs) to be identified which can be used in mapping. For example, nematode mutations can be mapped to SNPs and identified by genome resequencing and their function validated by RNAi. In addition, *H. bacteriophora* cis- and untranslated regulatory elements can be identified and used to facilitate expression of transgenes. These approaches can be used to learn how the nematode associates with symbiotic bacteria, what is the basis for dependency of these nematodes on symbiotic bacteria for reproduction and how do nematodes function as parasites? Therefore, the *H. bacteriophora* TT01 genome facilitates both basic and applied research on entomopathogenic nematodes.

## Materials and Methods

### Nematode Culture

An inbred line, M31e, self-fertilized 13 times, of *H. bacteriophora* TT01 strain originally isolated from Trinidad and Tobago [106] and kindly provided by Dr. Ann Burnell (NUI-Maynooth, Ireland), was thawed from cryopreserved stocks [24]. Axenic IJs were obtained by culturing the nematodes on strain *P. temperata* TRN16 that do not colonize IJs [26]. High molecular weight DNA was purified from first and second larval stages harvested from lawns of TRN16 grown previously for 18 h at 28°C on NA+chol (4 g nutrient agar, 1 g sodium pyruvate, 10 g agarose per liter with 2 ml 5 mg/ml cholesterol added after autoclaving). On average, 275 IJs were added to 100 mm lawns for efficient egg laying. Nematodes were washed off the lawns after 82–86 h with 10 ml of Ringer’s containing 0.1% triton X-100. Bacteria were removed by washing on a 10 **µ**m pore nylon filter and hermaphrodites removed by retention on a 30 **µ**m filter. Eggs were surface sterilized with 1% commercial bleach (Chlorox®), washed 3X in Ringer’s solution and allowed to hatch in Ringer’s solution containing 100 **µ**g/ml carbenicillin, 50 **µ**g/ml streptomycin, 30 **µ**g/ml kanamycin and 10 **µ**g/ml gentamicin overnight. A contaminant of *Stenotrophomonas maltophilia,* likely originating from a contaminated Ringer’s solution, was inadvertently sequenced along with the nematode. Approximately 3×10^6^ L1 nematodes were harvested from 1,000 cultures.

### Isolation of RNA

Nematode mRNA was isolated from mixed (L1–L4), adult and IJ stages grown on TRN16. The nematodes were obtained from the cultures with Ringer’s solution and bacteria removed by 3 washes with 15× Ringer’s solution in a 15 ml conical tube and centrifugation for 5 min at 2,000 rpm. The nematodes were frozen in liquid nitrogen, then Trizol reagent (Life Technologies) was added and incubated at 65°C. The IJs were freeze-thawed 3× in liquid nitrogen and at 65°C, before RNA was purified per manufacturer’s instructions. Polyadenylated RNA was purified using oligo(dT) cellulose columns, MicroPoly(A)Purist Kit (Life Technologies).

### cDNA Library Construction and Sequencing

The integrity of the mRNA was validated using the Bioanalyzer 2100 (Agilent Technologies) and yield determined via Nanodrop (Thermo Scientific). Two different methods were used for library construction:

1. The CloneMiner cDNA Library Construction Kit (Life Technologies) was utilized to generate non-radiolabeled cDNA according to the manufacturer’s specifications. A Biotin-attB2-Oligo(dT) primer was hybridized to mRNA. First strand cDNA was synthesized via SuperScript II Reverse Transcriptase. DNA polymerase I was utilized to generate the second strand of cDNA. attB1 adapters were ligated to the 5′ end of the cDNA. The cDNA was purified by column fractionation to remove residual adapters. Through site-specific recombination, attB-flanked cDNA was cloned directly into the pDONR-222 vector (Life Technologies). The ligations were transformed using the ElectroMax DH10B cells (Life Technologies). The transformed cells were spread on LB plates containing 50 **µ**g/mL kanamycin.
2. mRNA was used as the template for cDNA library construction using the Accuscript HF Reverse Transcriptase Kit (Agilent Technologies) and SMART primers (Life Technologies). PCR cycle optimization was performed to determine the threshold cycle number to minimally amplify full length cDNA products using the SMART primers and Clontech Advantage-HF 2 polymerase Mix (Clontech/Takara Bio). Library normalization was accomplished by using the Trimmer kit (Evrogen). PCR cycle optimization was performed with normalized cDNA to determine the threshold cycle number using the SMART primers and Clontech Advantage-HF 2 polymerase Mix previously mentioned. Finally, 5′ and 3′ adapter excision was performed by restriction exonuclease digestion using *Mme*I. The excised adapters were removed utilizing AMPure paramagnetic beads (Agencourt, Beckman Coulter Genomics). Two kinds of libraries were prepared for sequencing on ABI3730 and Roche/454 platforms.

For libraries intended for sequencing on ABI3730 platform, the final cDNA product was nebulized, end repaired (Lucingen), and size selected from a 0.8% SeaKem agarose TAE gel. The fraction was purified according to the manufacturer’s instructions in the QIAquick Gel Extraction (Qiagen) protocol and ligated into the pSMART HC-Kan vector system (Lucigen). Ligations were transformed using *E. coli* cells (Lucingen). The transformed cells were spread onto LB plates containing 50 **µ**g/mL kanamycin.

A 454 fragment library was constructed using GS DNA Library Preparation Kit (Roche) with the cDNA as outlined in the manufacturer’s protocol. Five microgram of cDNA was fragmented via nebulization. Fragmented cDNA was size selected with an AMPure bead (Agencourt, Beckman Coulter Genomics) cleanup, removing fragments less than 300 bp. The cDNA was end polished and ligated to 454 Titanium library adapters utilizing reagents from the Titanium General Library Kit (Roche). An AMPure (Agencourt) bead cleanup was performed to remove library adapter dimers and cDNA fragments less than 400 bp in length. The 454 library was immobilized with Strepavidin beads (-Roche) and single stranded with Sodium Hydroxide. The single stranded library was quantitated by a Quant-iT single stranded DNA assay using the Qubit fluorometer (Life Technologies) and the integrity validated using the BioAnalyzer 2100 (Agilent Technologies). The library fragments were immobilized onto DNA capture beads utilizing clonal amplification kits (Roche). The captured DNA library was emulsified and subjected to PCR in order to amplify the DNA template. The emulsion was chemically broken and the beads containing the DNA were recovered, washed, and enriched utilizing bead recovery reagents (Roche). The DNA library beads were loaded onto a PicoTiterPlate device and sequenced on the Genome Sequencer instrument using the GS FLX Titanium Sequencing Kit XLR70 (Roche).

### Genomic Library Construction and Sequencing

High molecular weight genomic DNA was isolated using a protocol kindly provided by Erich Schwartz, which was based on that of Andrew Fire’s lab with slight modifications from the R. Waterston lab and K. Kiontke [107]. The integrity of the genomic DNA was verified by comparing the intensity of *H. bacteriophora* to serial dilutions of lambda standards of known concentration on a 1.8% agarose gel stained with ethidium bromide. The yield was determined by a high sensitivity Quant-iT double stranded DNA assay using a Qubit fluorometer (Life Technologies). A 454 Titanium fragment library was constructed with 5 µg of genomic DNA as outlined in the manufacturer’s protocol. The genomic DNA was fragmented via nebulization and run on a 0.8% GTG Seakem agarose gel (Lonza) with ethidium bromide in 1× TAE buffer for a size selection of 500–800 bp. Fragmented DNA was isolated from the agarose gel using the QiaQuick Gel Extraction Kit (Qiagen). The size selected DNA was end polished and ligated to 454 Titanium library adapters utilizing reagents from the Titanium General Library Kit (Roche). An AMPure (Agencourt) bead cleanup was performed to remove library adapter dimers and DNA fragments less than 400 bp in length. The 454 library was immobilized with Strepavidin beads (Roche) and single stranded with sodium hydroxide. The single stranded library was quantitated by a Quant-iT single stranded DNA assay using a Qubit fluorometer (Life Technologies) and the integrity validated using the BioAnalyzer 2100 (Agilent Technologies). The library fragments were immobilized onto DNA capture beads utilizing clonal amplification kits. The captured DNA library was emulsified and subjected to PCR in order to amplify the DNA template. The emulsion was chemically broken and the beads containing the DNA were recovered, washed, and enriched utilizing bead recovery reagents. The DNA library beads were loaded onto a PicoTiterPlate device and sequenced on the Genome Sequencer instrument using the GS FLX Titanium Sequencing Kit XLR70 (Roche).

### Genome Assembly

The genome sequences from fragments, 3 kb insert from plasmid libraries and end sequencing of bacterial artificial clone libraries were generated at an estimated 26-fold sequence coverage. All sequenced reads were attempted in de novo assembly using the Celera assembler v. 6.0. The assembly was submitted to GenBank genome database under accession number ACKM00000000.

### Genome Annotation

The scaffolds were masked for repeats using RepeatMasker version 3.3 [108]. Transfer RNA coding genes were predicted using tRNAscan-SE [109]. To identify microRNA, other non-coding RNA, and regulatory elements, Rfam [110] covariance models were searched using Inferno program [111], [112] with an E value cutoff of 1e-8 after adjusting to the size of the genome. Protein-coding genes were predicted with gene prediction programs of SNAP [113], AUGUSTUS [114]–[116], GlimmerHMM [117], and GeneMark [118]. The results were integrated with other evidence, including the mapping results of ESTs generated by cDNA sequencing with sim4 and sequence similarity to proteins in GenBank non-redundant (nr) database and WormBase WS220 release, by JIGSAW program [119] with linear combiner option. Gene models with in-frame stop codons were considered erroneous and therefore removed. Protein domains in the predicted protein-coding genes were predicted by searching Pfam [120] using the HMMER program [82] with an E value threshold of 1e-4. For comparison, the same prediction parameters were used to predict Pfam domains in other nematodes. A domain richness index for each domain in each nematode was calculated by dividing the number of that domain with the total number of protein sequences in that nematode species. The program T statistics was used to compare the domain richness indices among nematodes. *H. bacteriophora* protein sequences were assigned Gene Ontology terms by the Blast2GO program [121] based on the BLASTp results against the SwissProt database with an E value cutoff of 1e-10. The orthologous sequences among *H. bacteriophora*, *C. elegans*, *C. briggsae*, *C. japonica*, *C. remanei*, *C. brenneri*, *Brugia malayi*, *Meloidogyne hapla*, *M. incognita*, and *Pristionchus pacificus* were identified using the orthoMCL program [101] on the predicted protein sequences from the genomes. *H. bacteriophora* protease/peptidases were predicted based on sequence similarity search of the sequences in MEROPS database Release 9.5 [122].

### Estimation of Divergence Time between *H. bacteriophora* and *C. elegans*

We obtained a set of 350 orthologs common to *H. bacteriophora*, *C. elegans*, *Anopheles gambiae* (AgamP3.4 release from VectorBase), and *Homo sapiens* (Ensembl release 55) based on the prediction results of orthoMCL [101]. For each ortholog set, the protein sequences were aligned using ClustalW2 [123], followed by reverse translation to their original transcript sequences that were obtained from the same respective databases as the protein sequences. After conversion to PHYLIP format, the alignments were used to estimate genetic distances among the taxa using the DNADIST program in PHYLIP (PHYLogeny Inference Package; [124]. A phylogenetic tree was then built using the PHYLIP neighbor-joining algorithm NEIGHBOR with human as the outgroup taxon. The sequence alignment and the rooted neighbor-joining tree were used to estimate divergence times using the MCMCTREE program in PAML (Phylogenetic Analysis by Maximum Likelihood [125]). We used 800–1000 MYA (million years ago) as the divergence time of nematodes and insects [91].

### Gene Ontology Enrichment and Metabolic Pathway Comparison

*H. bacteriophora* protein sequences were assigned Gene Ontology (GO) terms by the Blast2GO program [121] based on the BLASTP results against SwissProt database with an E value cutoff of 1e-10. In comparison, proteins from the other 9 nematode genomes underwent the same analysis using the same programs and databases. The pair-wise GO enrichment using *H. bacteriophora* sequences as the reference was done using the GOSSIP program [126]. The KEGG (Kyoto Encyclopedia of Genes and Genome) Ontologies (KO) in the metabolic pathways were assigned using Blast2GO program [121] for the four nematode species being compared.

### Ethics Statement

This study did not involve any human or vertebrate subjects.

## Supporting Information

Table S1 **Predicted gene models in** ***Heterorhabditis bacteriophora*** **genome.**
(XLSX)

Table S2 **Sequence similarity of conceptually translated** ***H. bacteriohora*** **proteins to** ***C. elegans*** **proteins in Wormbase release W220.**
(XLSX)

Table S3 **Comparison of Pfam domains predicted in the proteins of 10 nematode species in this study.**
(XLSX)

Table S4 **Predicted mariner DNA motifs in** ***H. bacteriophora*** **genome.**
(XLSX)

Table S5 **Predicted mariner DNA motifs in** ***C. elegans*** **genome using the same parameters as the ones used to generate results in Table S4.**
(XLSX)

Table S6 **Predicted non-protein-coding RNA in** ***H. bacteriohora*** **genome.**
(XLSX)

Table S7 **Predicted tRNA genes in** ***H. bacteriophora*** **genome.**
(XLSX)

Table S8 **Predicted microsatellite loci in** ***H. bacteriophora*** **genome.**
(XLSX)

Table S9 **Predicted secretome in** ***H. bacteriophora*** **genome.**
(XLSX)

Table S10 **Comparison of gene ontology terms that were assigned to genes in the 10 nemtode species included in this study.**
(XLSX)

Table S11 **Comparison of Kyoto Encyclopedia of Genes and Genomes (KEGG) pathways predicted in the 10 nematode species included in this study.**
(XLSX)

Table S12 **Comparison of the metabolism pathways in KEGG Ontologies in the 10 nematode species included in this study.**
(XLSX)

Table S13 **The list of the IDs of orthologous sequences in the 10 nematode species included in this study.**
(XLSX)
